# Prospective clinical validation of the Empatica EmbracePlus wristband as a reflective pulse oximeter

**DOI:** 10.3389/fdgth.2023.1258915

**Published:** 2023-12-04

**Authors:** Giulia Gerboni, Giulia Comunale, Weixuan Chen, Jessie Lever Taylor, Matteo Migliorini, Rosalind Picard, Marisa Cruz, Giulia Regalia

**Affiliations:** ^1^ Empatica, Inc., Cambridge, MA, United States; ^2^ MIT Media Lab, Massachusetts Institute of Technology, Cambridge, MA, United States

**Keywords:** reflective pulse oximeter, wearable device, wrist photoplethysmography, FDA clearance, blood oxygen saturation, respiratory disease

## Abstract

**Introduction:**

Respiratory diseases such as chronic obstructive pulmonary disease, obstructive sleep apnea syndrome, and COVID-19 may cause a decrease in arterial oxygen saturation (SaO_2_). The continuous monitoring of oxygen levels may be beneficial for the early detection of hypoxemia and timely intervention. Wearable non-invasive pulse oximetry devices measuring peripheral oxygen saturation (SpO_2_) have been garnering increasing popularity. However, there is still a strong need for extended and robust clinical validation of such devices, especially to address topical concerns about disparities in performances across racial groups. This prospective clinical validation aimed to assess the accuracy of the reflective pulse oximeter function of the EmbracePlus wristband during a controlled hypoxia study in accordance with the ISO 80601-2-61:2017 standard and the Food & Drug Administration (FDA) guidance.

**Methods:**

Healthy adult participants were recruited in a controlled desaturation protocol to reproduce mild, moderate, and severe hypoxic conditions with SaO_2_ ranging from 100% to 70% (ClinicalTrials.gov registration #NCT04964609). The SpO_2_ level was estimated with an EmbracePlus device placed on the participant's wrist and the reference SaO_2_ was obtained from blood samples analyzed with a multiwavelength co-oximeter.

**Results:**

The controlled hypoxia study yielded 373 conclusive measurements on 15 subjects, including 30% of participants with dark skin pigmentation (V–VI on the Fitzpatrick scale). The accuracy root mean square (*A*_rms_) error was found to be 2.4%, within the 3.5% limit recommended by the FDA. A strong positive correlation between the wristband SpO_2_ and the reference SaO_2_ was observed (*r* = 0.96, *P* < 0.001), and a good concordance was found with Bland–Altman analysis (bias, 0.05%; standard deviation, 1.66; lower limit, −4.7%; and upper limit, 4.8%). Moreover, acceptable accuracy was observed when stratifying data points by skin pigmentation (*A*_rms_ 2.2% in Fitzpatrick V–VI, 2.5% in Fitzpatrick I-IV), and sex (*A*_rms_ 1.9% in females, and 2.9% in males).

**Discussion:**

This study demonstrates that the EmbracePlus wristband could be used to assess SpO_2_ with clinically acceptable accuracy under no-motion and high perfusion conditions for individuals of different ethnicities across the claimed range. This study paves the way for further accuracy evaluations on unhealthy subjects and during prolonged use in ambulatory settings.

## 1. Introduction

Respiratory diseases like chronic obstructive pulmonary disease (COPD), asthma, obstructive sleep apnea syndrome (OSA), and COVID-19 account for a significant burden of disease (1, 2). COPD, associated with persistent and progressive respiratory symptoms (3), has a global prevalence of 3.9%, and, in 2019, was the third leading cause of death worldwide (1, 3). Asthma is caused by inflammation and narrowing of the small airways in the lungs (4) and affects approximately one in five individuals (5). In the U.S., 26% of individuals between 30 and 70 years suffer from OSA, which manifests in complete or partial upper airway obstruction (6). Over the past three years, clinical COVID-19 associated with the CoV-2 coronavirus has also resulted in substantial morbidity and mortality, causing over 6 million deaths worldwide to date (7). The common pathophysiology underlying these respiratory diseases is impaired gas exchange, which depending on the severity and duration of impairment, can result in hypoxemia (1–8) and associated clinical signs and symptoms ranging from headaches and dyspnea to cellular hypoxia, organ failure, and death in extreme cases (9, 10).

Since arterial oxygen saturation (SaO_2_) below the physiological 95%–100% range can be suggestive of respiratory pathology but is not always associated with apparent symptoms such as dyspnea, monitoring of blood oxygenation in individuals at risk of new or worsening hypoxemia is an important tool to enable early identification of clinical decompensation, and to inform timely decision-making about interventions including hospitalization, ICU admission, and supplemental oxygen therapies (e.g., mechanical ventilation) (10). The clinical standard and most accurate method for assessing SaO_2_ is co-oximetry, which requires invasive measurements to detect SaO_2_ values from blood samples. Co-oximetry allows only intermittent and point-in-time measurements of SaO_2_, which are not compatible with continuous monitoring in ambulatory settings and are thus not appropriate for analysis of longitudinal trends in the SaO_2_ (9, 11, 12).

Pulse oximetry is a non-invasive modality for monitoring SaO_2_ estimations by measuring the peripheral oxygen saturation (SpO_2_) using photoplethysmography (PPG) technology. PPG-based fingertip pulse oximeters have become the de facto standard for assessing SpO_2_ in hospitalized patients due to their non-invasiveness, flexibility (13, 14), and ability to meet clinical accuracy thresholds established by the ISO 80601-2-61:2017 standard and the US Food & Drug Administration (FDA) guidance on pulse oximetry validation (15, 16), which were established in 2017 and in 2013, respectively. Recently, PPG-based sensors have been increasingly adopted also in mobile digital health technologies for remote monitoring applications, as part of the modern paradigm shift of clinical care and clinical research to home-centered healthcare and decentralized clinical trials (11, 13, 14, 17–19). Among various physiological parameters, SpO_2_ measurements in ambulatory settings are now provided by several wireless commercial devices, with different degrees of validation (20) and different form factors, including devices worn on the upper arm (19, 21), on the chest (19, 22), on the ear (23), or on the wrist (11, 18, 19, 24–31).

Among wearables, wrist-worn devices have several advantages thanks to their portability, comfort, easy acceptance, and non-stigmatization, resulting in high compliance (32). However, getting SpO_2_ estimations from the wrist is challenging given lower arterial blood perfusion and lower signal-to-noise ratio resulting from multiple tissue scattering in the dorsal wrist compared to other locations (e.g., finger, ear) (14, 33, 34). The number of wrist-worn devices that received clearance from the FDA for PPG-based technologies remains quite low (27–31). These wrist-worn devices have been cleared based on clinical evidence abiding by the FDA/ISO standardized protocols which require a minimum of 200 data points each for 10 or more healthy volunteers that vary in age, sex, and skin pigmentation, including 2 darkly pigmented subjects or 15% of study pool, whichever is greater (15, 16). To date, however, details of validation studies supporting clearance of these devices have not been widely published (26, 35).

Recently, the publication of post-market data related to the real-world use of cleared pulse oximeters has raised questions about the performance of these devices, particularly in individuals with darker skin tones (36), leading to increased scrutiny by the FDA and the scientific and clinical community (37, 38). This issue was documented based on data from finger pulse oximeters using transmissive technology, which has a superior signal-to-noise ratio compared to reflective sensors. Thus, a higher impact of skin color could be expected on devices with reflective PPG technology, since scattering due to melanin would further increase the scattering seen at baseline in wrist-worn devices (39).

Given the paucity of published validation studies for clinical data from wrist-worn wearables, and recently raised concerns that existing pulse oximeters may not perform accurately in racial minorities (36–39), it is critical to rigorously test new wrist-worn pulse oximeters, especially to verify their accuracy and reliability across different skin pigmentations. This work contributes to filling this gap by reporting the prospective validation of the SpO_2_ measurements computed by a wrist-worn medical device, i.e., the EmbracePlus wristband, on a pool of subjects enriched for individuals with darker skin pigmentation (30% of study participants). This device and its associated monitoring platform recently received clearance from the FDA to allow healthcare professionals to monitor SpO_2_ in no-motion and high perfusion conditions in ambulatory individuals aged 18 years and older in home healthcare settings (40). This work details the methods and the results of the comparison between EmbracePlus SpO_2_ measurements and gold standard measures of SaO_2_, performed during a controlled hypoxia study following the ISO 80601-2-61:2017 standard (15) and the FDA guidance (16). The validation presented herein complies with recently published suggestions to increase the statistical robustness of results, transparency, and understanding of limitations of pulse oximetry. Moreover, this study reports individual subjects accuracy levels, subgroup analysis by different skin pigmentation on a dataset that doubles the representation of darkly pigmented individuals with respect to FDA guidance (16) and clinically relevant information about outliers (41).

## 2. Methods

### 2.1. PPG principles

**Figure 1:**
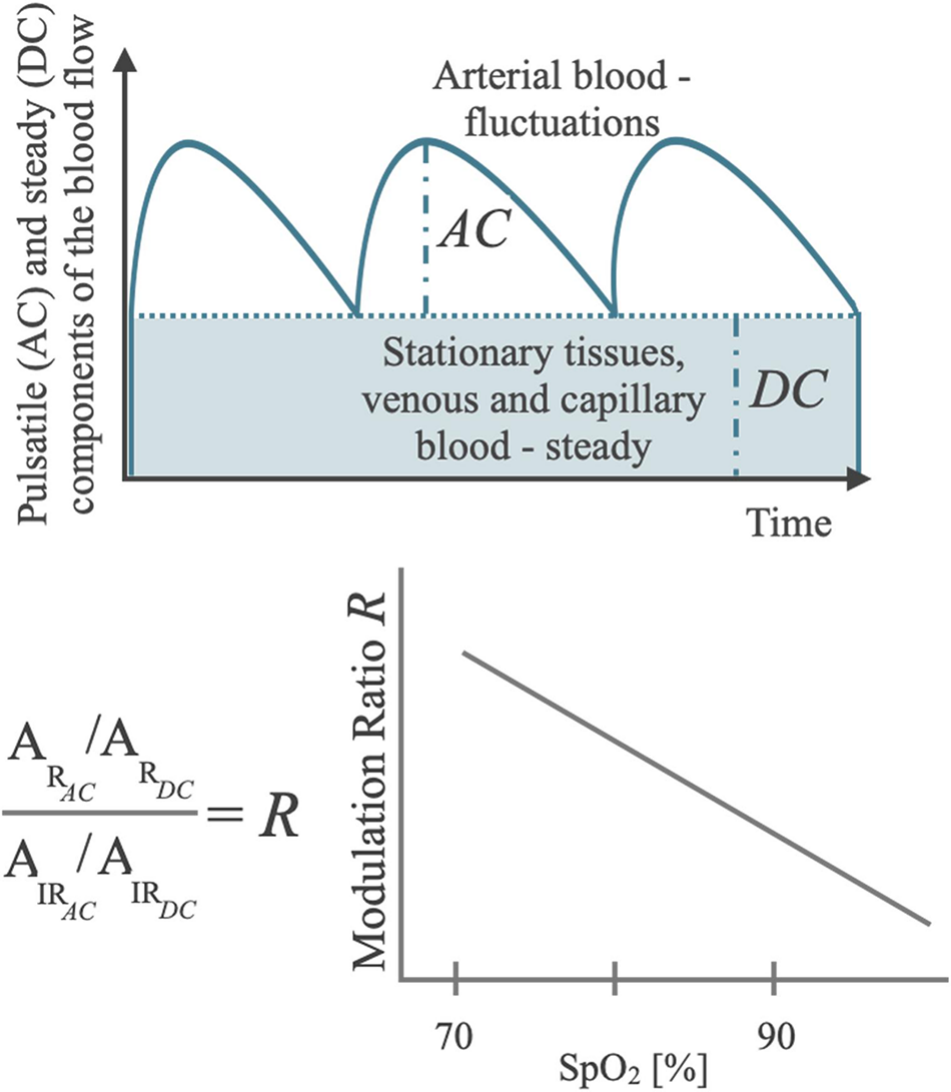
Schematic diagram of the basic principle of a pulse oximeter showing the components of the blood flow in the tissues in relation to the optical metrics of interest for SpO_2_ computation (AC and DC), with the diagram of a typical regression function that maps AC and DC composite metrics into a SpO_2_ Reading.

The SpO_2_ algorithm is based on PPG technology and harnesses two principles: (i) the different absorption spectra of the oxygenated (HbO_2_) and the deoxygenated (HbH) hemoglobin, and (ii) the presence of a pulsatile arterial blood flow (42, 43). The former is exploited to compute the concentrations of HbO_2_ and HbH by illuminating the skin with two different wavelengths, typically red (∼660 nm) and infrared (∼940 nm). Indeed, HbO_2_ absorbs more infrared light while the red light more easily passes through, whereas HbH absorbs more red light and allows more infrared light to pass through (42–44). The relative amount of red and infrared light that is reflected towards a photodetector (PD) after being partially absorbed by the arterial blood can be used to ultimately estimate the proportion of the hemoglobin bound to oxygen and therefore the SpO_2_ level, based on the Lambert-Beer law of absorbance (45). The second principle leverages the respective inherent contractility of arteries and veins. During each cardiac cycle, the arterial blood volume increases during systole and decreases during diastole, leading to fluctuations in the absorbed red and infrared light, which form the pulsatile (AC) component. By contrast, light that is reflected and reaches the PD from blood volume in veins and capillaries or non-vascular tissues presents a relatively stable, non-pulsatile component, which forms the steady (DC) component (33, 44). The perfusion ratio (*R*) (i.e., the ratio of AC/DC of red and infrared PPG) is used to derive the SpO_2_-estimation using an empirical calibration function derived from the relationship between *R* and SpO_2_, which is obtained in experimental conditions through a stable and controlled hypoxic condition spanning the claimed measurement range (e.g., 70%–100% SpO_2_) (Figure 1) (44, 46).

### 2.2. EmbracePlus SpO_2_ algorithm

**Figure 2:**
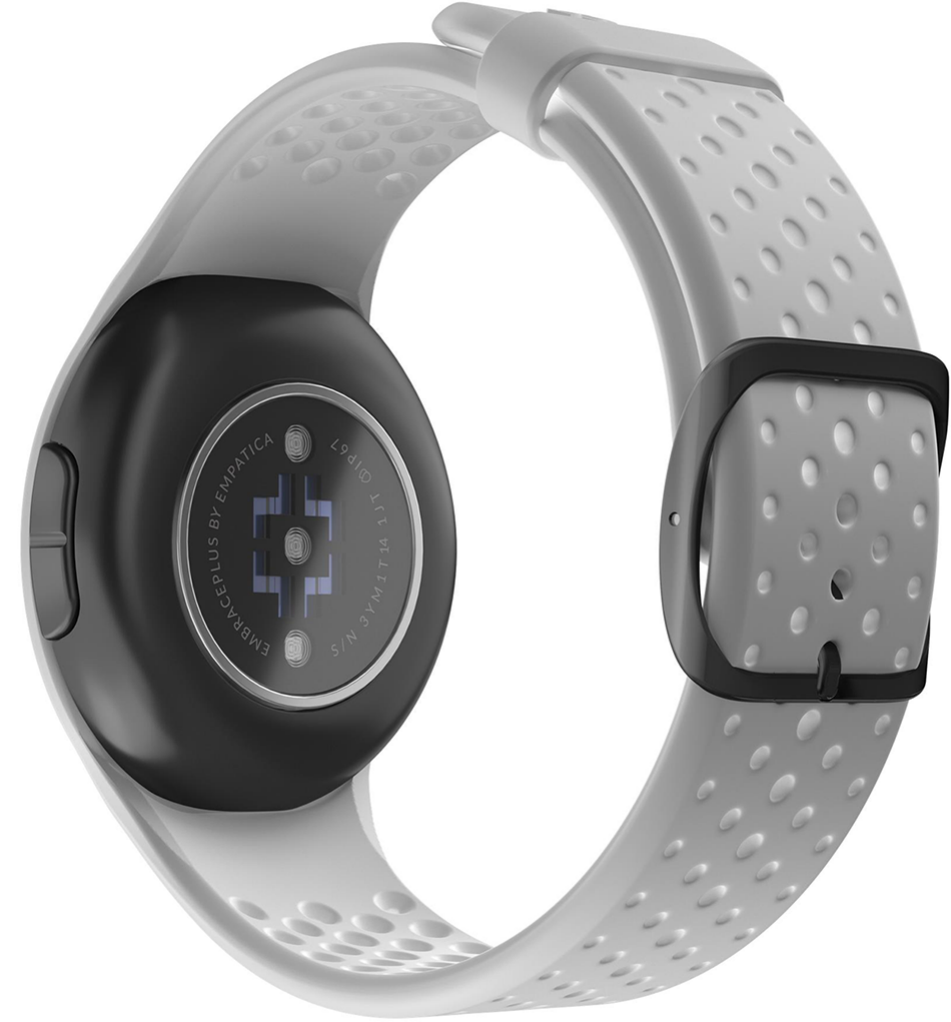
Back view of the EmbracePlus wristband with the reflectance PPG sensor embedded in the device.

The inputs of the SpO_2_ algorithm are data obtained illuminating the skin with green, red, and infrared-light PPG sensors and data recorded by three-axis accelerometry (ACM) sensors embedded in the EmbracePlus device (Figure 2). The algorithm analyzes the red and infrared PPG signals to extract the amplitude of the AC pulsatile component and of the DC baseline component (44), using the green PPG to support the detection of AC. Using the Lambert-Beer model (47), these metrics can be used to estimate the SpO_2_ value using a calibration model previously obtained during the training phase, which harnessed data from a controlled hypoxia calibration study and from real-life data. The data used to develop the algorithm were completely independent from the datasets used for the prospective clinical validation presented in this work, i.e., the development and validation datasets did not contain data from the same subjects.

Each value of SpO_2_ is estimated on a 10 s rolling window offset by 1 s. A missing SpO_2_ value indicates that the algorithm does not have enough confidence to compute an output. A low level of confidence is principally driven by the user not wearing or improperly wearing the device, or by the presence of low-quality raw sensor data (e.g., during motion conditions or low perfusion). Indeed, reflective PPG positioned on the dorsal portion of the wrist has been associated with a lower signal-to-noise ratio compared to the transmissive PPG sensors (26, 48, 49). This effect might be due to both the sensor placement, since only a small portion of the light is reflected and reaches the PD, and to the sensor design accounting for multiple scattering through the skin layers and movement artifact contamination associated with the probe contact pressure (49). In addition, blood perfusion is lower on the back of the wrist as compared to the finger (26). Thus, since SpO_2_ measured at the wrist can be prone to lower signal quality and is sensitive to movement contamination, the EmbracePlus utilizes an automated data rejection mechanism (i.e., quality index) based on PPG and ACM to detect a signal quality threshold for which it is possible to estimate trustable SpO_2_, discarding inconclusive SpO_2_ measurements in the condition of low perfusion, movement, and low signal quality (34, 50).

The algorithm involves signal processing steps in time-domain (e.g., linear filtering, cross-correlation) and frequency-domain (e.g., spectral analysis), and a linear regression model which are compatible with an online implementation where output SpO_2_ values are estimated over consecutive 10 s windows offset by 1 s. No “future-time” data point is used to estimate SpO_2_ in a given 10 s window.

### 2.3. Recruitment

The protocol received IRB approval (Laurel Heights Committee—approval number 10-00437; Clinicaltrials.gov registration #NCT04964609) to test the accuracy of the EmbracePlus SpO_2_ measurements during mild, moderate, and severe hypoxia. A single-center and interventional clinical study was conducted on 16 healthy participants in a laboratory at the University of California San Francisco between June 2021 and January 2023. Healthy male and female subjects between the ages of 18 and 55 years were recruited, excluding current smokers, women who were pregnant, lactating, or trying to get pregnant, and participants with obesity (body mass index, BMI > 30 kg/m^2^) or who had an injury, deformity, tattoos, or other physical abnormality at the sensor sites. Exclusion criteria also included participants with serious systemic illnesses, and those who use continuous positive airway pressure, have unacceptable collateral circulation, or any other condition which in the investigators’ opinion would make them unsuitable for the study.

Participants were primarily selected to represent a heterogeneous population in terms of skin tone which was assessed by the Clinical Coordinator at Hypoxia Lab based on the Fitzpatrick scale for skin pigmentation assessment, recruiting 30% of participants with skin tone classified as Fitzpatrick V or VI, i.e., doubling FDA requirements for inclusion of individuals with dark skin (16). Moreover, the recruitment process aimed at including a participant pool with varying ages, BMI, and sex balance. The sample size was selected according to ISO 80601-2-61:2017, which recommends including at least 200 data points from at least 10 subjects (15).

### 2.4. Study design

The test was conducted in accordance with ISO standards (15) and FDA guidance (16) for SpO_2_ testing, which require evaluation against a SaO_2_ reference measurement ranging from 70% to 100% during a controlled desaturation protocol.

**Figure 3:**
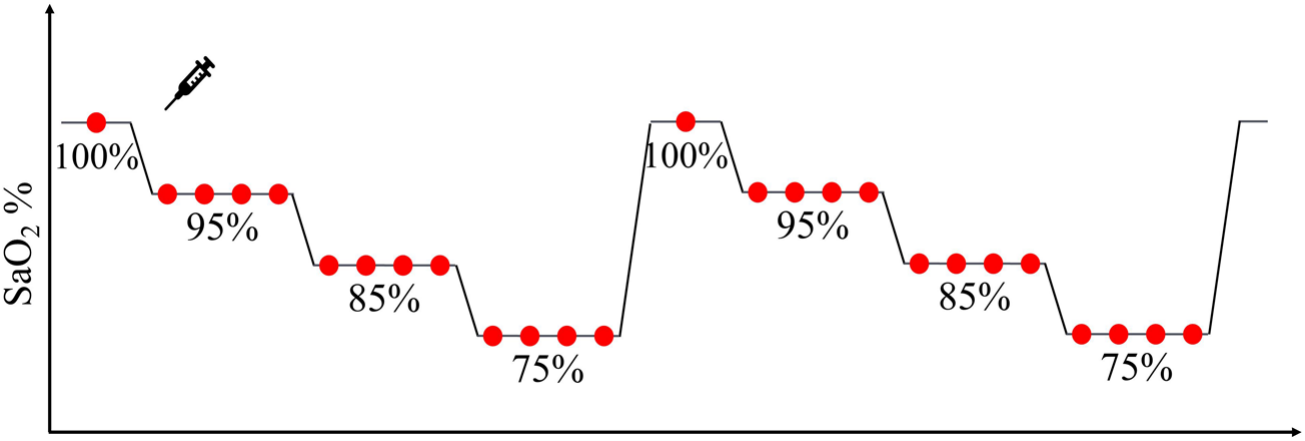
Graphical representation of each desaturation run, consisting of a stepwise decrease of blood oxygen through stable plateaus during which a minimum of two blood samples (red circles) were taken to measure reference SaO_2_.

Each participant was placed in a comfortable semi-recumbent position for approximately 45 min and asked to remain still while breathing a mixture of gas through a mouthpiece while supervised by a medical monitor. Participants’ hands and arms were maintained at ambient room temperature during data collection. Data were collected from an EmbracePlus wristband and two FDA-cleared finger-tip pulse oximeters (Masimo Rad-5 by Masimo Corp and Nellcor N-595 by Nellcor Puritan Bennett Inc.) which were used to monitor the hand perfusion and synchronize the reference SaO_2_ data with the EmbracePlus data. All the devices were positioned according to their respective instructions for use. The non-dominant radial artery of each participant was connected to an arterial line (a 22-gauge catheter) to draw blood samples on which the SaO_2_ was analyzed by a laboratory multiwavelength co-oximeter (ABL-90 blood gas analyzer, Radiometer Medical ApS) and used as ground truth for the SpO_2_ algorithm evaluation. Then, each participant underwent two desaturation runs consisting of a stepwise decrease of the oxygen concentration in the inspired gas mixture, as illustrated in Figure 3.

At the beginning of each ramp, a baseline blood sample was collected at room air. Approximately 10 s later, the inspired oxygen was progressively reduced to reach the next SpO_2_ plateau level, identified by a stable level of oxygen saturation between the reference finger pulse oximeters. At each target SpO_2_ plateau, two to four blood samples were collected approximately 30 s apart, and within 10 s from the conclusion of each plateau, inspired oxygen was progressively changed again to reach the next SpO_2_ target level. The target SpO_2_ at each run was chosen to allow an even sampling within the [70%–100%] SaO_2_ range. Every participant underwent a maximum of 6 plateaus in each ramp before being exposed to a high oxygen saturation level (100% O_2_) by breathing oxygen-enriched air for 2 min. The collected blood samples (approximately 20–26 samples per participant) were immediately analyzed with the co-oximeter to measure the reference SaO_2_.

### 2.5. Data handling

Prior to the analysis, periods corresponding to EmbracePlus recording failure (i.e., missing raw data) and out-of-range values from the reference SaO_2_ measurement device (i.e., SaO_2 _< 67%) were identified and removed. EmbracePlus and finger pulse oximeter data were aligned using the pulse rate series estimated on EmbracePlus PPG and the pulse rate series logged by the finger pulse oximeters. The procedure was blind, and it was performed without the knowledge of SaO_2_ values. However, since the timestamps of the reference SaO_2_ were already synchronized with the finger pulse oximeter data, this procedure allowed automatic alignment with the SpO_2_ algorithm outputs and the reference SaO_2_.

EmbracePlus continuous SpO_2_ data were analyzed to select the values associated with each SaO_2_ reference reading. The median SpO_2_ value inside a 10 s window within the plateau associated with each blood sample was computed and used to determine the paired value for performance evaluation. The window of SpO_2_ values was adjusted to fit a segment with good-quality EmbracePlus data within the selected plateau, to discard the effect of involuntary movements occurring during the procedure (e.g., when the sample was taken).

### 2.6. Statistical analysis

The primary endpoint of both studies was the accuracy root mean square (*A*_rms_), which is a combination of the systematic and random components of error, computed as the root-mean-square differences between the algorithm output (SpO_2*i*_) and the reference (SaO_2*i*_) (Equation 1), where *N* is the total number of data points. Data from all the subjects were pooled together for *A*_rms_ computation to verify the primary effectiveness endpoint. Moreover, individual *A*_rms_ values were computed on each subject's data. Only the data pairs with evaluable values for both the EmbracePlus and the reference device were used.

(1) $$A_{\mathrm{rms}}=\sqrt{\frac{\sum_{i=1}^{N}{\left(\mathrm{Sp}O_{2i}-\mathrm{Sa}O_{2i}\right)}^{2}}{N}}$$

According to FDA guidelines (16), a passing result required an *A*_rms_ ≤ 3.5% across the fully tested range under no-motion conditions, computed pooling the data points collected from all subjects. This threshold specifies that approximately two-thirds of the device measurements fall within ±3.5% of the reference measurement.

As additional performance measures, the mean bias (i.e., the average of the difference between SpO_2_ and SaO_2_) and the mean absolute error (MAE) (i.e., the average of the absolute difference between SpO_2_ and SaO_2_) were computed. A Bland–Altman analysis was performed on all the data points by plotting SaO_2_ versus error (SpO_2_—SaO_2_) with linear regression fit and upper 95% and lower 95% limits of agreement (LoAs) corrected for repeated measurements, indicating the error boundary where approximately 95% of data points fall (51). Additionally, a correlation analysis with linear regression fitting was performed on the pooled data to evaluate the correlation between the EmbracePlus SpO_2_ and the reference SaO_2_ values. Performance metrics were also evaluated on sex and skin-tone subgroups separately, namely on female and male subjects and on individuals with dark (Fitzpatrick class V and VI) and light skin pigmentation (Fitzpatrick classes I to IV). Furthermore, to investigate possible differences in the SpO_2_ estimation error between successive desaturation ramps, a mixed effect model was performed with the subject ID as a random effect and the desaturation ramp ID as fixed effect. An ANOVA was then performed to test the hypothesis that the coefficient representing the fixed-effect term is 0 (*F*-test with significance level at 0.05).

## 3. Results

The 16 participants in this study included 8 men and 8 women aged 18–43 years with various skin tones. Table 1 and Table 2 report the demographic summary and listing of the participants, respectively. No undesirable effects or adverse events were reported during the study.

**Table 1:** Summary of demographic characteristics of the study participants (*n* = 16).

Sex	Male	50% (8/16)
	Female	50% (8/16)
Skin tone [Fitzpatrick scale]	II	18.75% (3/16)
	III	18.75% (3/16)
	IV	31.25% (5/16)
	VI	31.25% (5/16)
Age [years]	Mean ± SD (*n*)	28.2 ±7.3 (16)
	Median	26
	Range (min, max)	(18, 43)
BMI [lb*703]/[in^2^]	Mean ± SD (*n*)	23.7 ± 2.8 (16)
	Median	24.1
	Range (min, max)	(17.5, 28.4)

SD, standard deviation; BMI, body mass index; lb, pound; in, inches.

**Table 2:** Demographic listing of the study participants.

Subject ID	Sex	Age [years]	BMI [lb*703]/[in^2^]	Ethnicity	Skin tone [Fitzpatrick scale]
1	Male	26	28.4	Caucasian	III
2	Male	21	17.5	Caucasian	II
3	Female	20	20.0	Caucasian	II
4	Female	42	20.8	Caucasian	III
5	Male	33	24.3	Caucasian	IV
6	Male	27	22.2	Asian	IV
7	Female	26	23.8	Middle eastern	IV
8	Male	31	23.3	Caucasian	III
9	Male	26	24.5	African American	VI
10	Male	26	25.1	Hispanic	IV
11	Female	27	21.7	Caucasian	II
12	Female	25	23.1	Other/Multiethnic	IV
13	Female	43	27.1	African American	VI
14	Female	18	25.7	African American	VI
15	Male	37	25.8	African American	VI
16	Female	23	25.6	African American	VI

**Figure 4:**
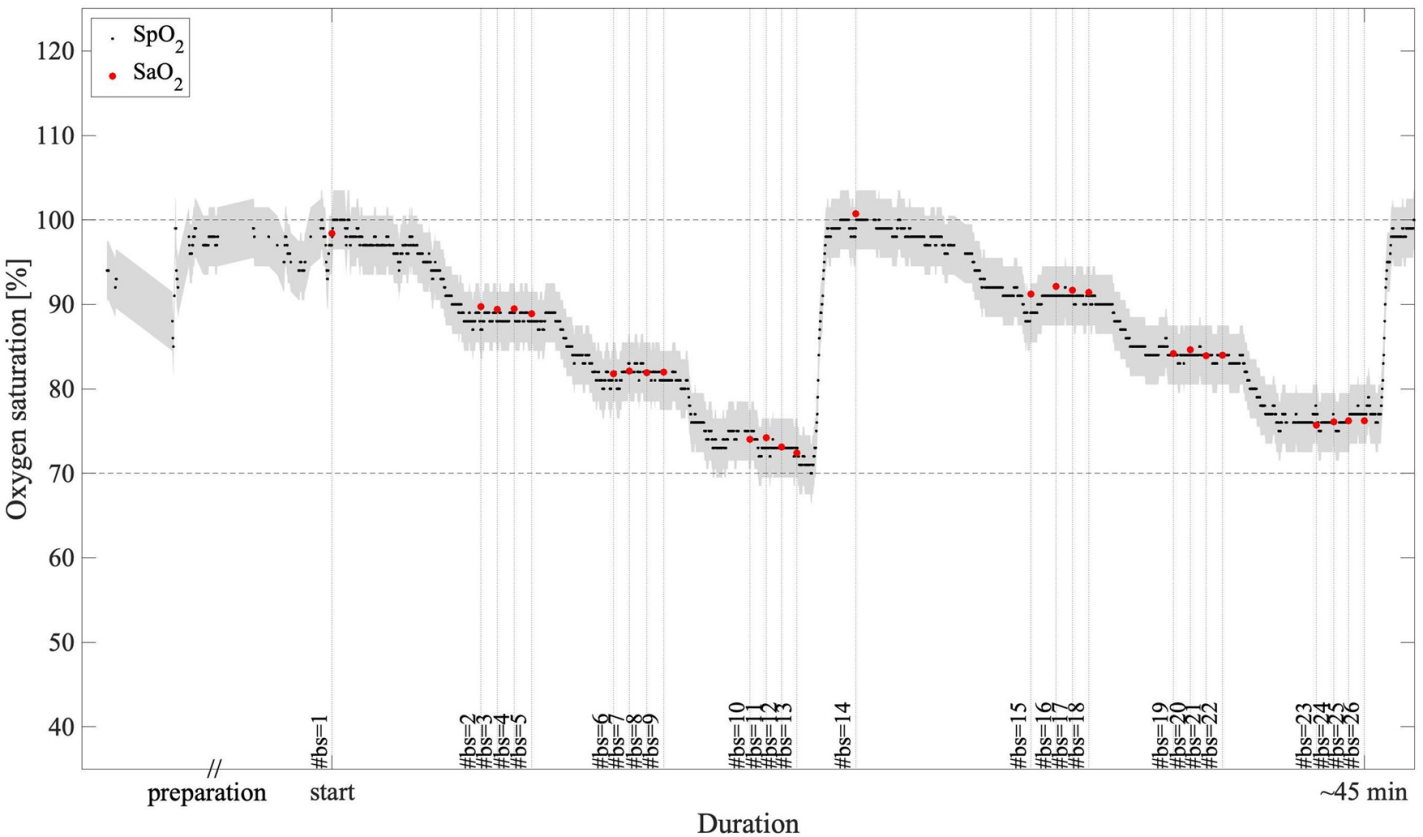
An example of SpO_2_ measurements during the two desaturation ramps: in black, the 1 s EmbracePlus SpO_2_ outputs; in red, the SaO_2_ samples; in grey, the clinical acceptance boundaries of ±3.5% centered on the EmbracePlus SpO_2_ outputs; #bs, number of the blood sample corresponding to each SaO_2_ measure.

Data from all subjects were included in the analysis, for a total of 398 samples of paired reference SaO_2_ and EmbracePlus SpO_2_ measurements. The recorded data did not include any missing EmbracePlus data or data affected by sensor issues. Out of the 398 blood samples, 1 sample from subject #6 could not be used for performance computation due to a missing timestamp for the reference SaO_2_. Out of the remaining 397 samples, the quality index embedded in the SpO_2_ algorithm automatically classified 24 samples collected on one subject (i.e., subject #2) as inconclusive, because of motion or low-quality PPG data, possibly related to the high level of stress experienced by the subject during the desaturation procedure, which was excluded in the performance assessment. Consequently, 373 SpO_2_ and reference SaO_2_ pairs from 15 subjects were used for the accuracy computation. An example of the EmbracePlus SpO_2_ trace during the ramps, superimposed with the SaO_2_ samples is reported in Figure 4. The measurements were drawn from a female subject classified as Fitzpatrick VI by skin tone.

The reference SaO_2_ showed a median value of 86.9% on the analyzed data, ranging from 68.1% to 100%. The paired measurements by the SpO_2_ algorithm showed a median value of 87% and ranged from 67% to 100%. The pooled *A*_rms_ was 2.4%, the bias 0.05%, the MAE 1.82%, and the upper and lower 95% LoAs were 4.8% and −4.7%, respectively (Table 3). Three levels of oxygen saturation were analyzed (i.e., SaO_2 _< 80%, 80% ≤ SaO_2 _< 90%, and SaO_2 _≥ 90%), on which pooled *A*_rms_ of 3.2%, 1.9%, and 2.2% and pooled bias values of 1.6%, 0.3%, and −1.2% were observed, respectively. In each SaO_2_ decile, pooled MAE was equal to 2.59%, 1.43%, and 1.68% and upper and lower 95% LoAs were ranging from −4.9% to 7.3% (Table 3). Additionally, Table 4 reports the individual *A*_rms_, bias, and MAE on the full SaO_2_ range together with the number of samples for each subject, which ranged from 20 to 26. Thirteen subjects (87%) demonstrated an A_rms_ lower than 3.5%.

**Table 3:** Distribution of the conclusive measurements collected by the EmbracePlus wristband and performance of the SpO_2_ algorithm in terms of *A*_rms_, bias and MAE for different SaO_2_ ranges.

Range of the reference SaO_2_	Number of paired samples [*n*]	Number of paired samples [%]	*A*_rms_ [%]	Bias [%]	MAE [%]	Upper 95% LoA [%]	Lower 95% LoA [%]
[67 100]%	373	100.0	2.4	0.05	1.82	4.8	−4.7
[67 80)%	93	24.9	3.2	1.6	2.59	7.3	−4.2
[80 90)%	138	37.0	1.9	0.3	1.43	4.0	−3.4
[90 100]%	142	38.1	2.2	−1.2	1.68	2.5	−4.9

**Table 4:** Performance of the SpO_2_ algorithm in terms of *A*_rms_, bias and MAE for each individual study participant.

Subject ID	*A*_rms_ [%]	Bias [%]	MAE [%]	*N*
1	2.0	1	1.6	20
3	1.8	0.5	1.5	25
4	1.8	−0.8	1.5	25
5	3.6	−0.5	3	25
6	3.0	1.7	2.3	24
7	2.3	−1.2	2.0	25
8	2.6	−0.5	2.1	25
9	4.2	3.4	3.4	25
10	2.8	0.6	2.5	25
11	2.5	−0.7	2.0	25
12	1.7	0.4	1.3	25
13	1.3	−0.7	1.1	26
14	2.2	−1.3	1.6	26
15	0.9	−0.4	0.7	26
16	0.9	−0.4	0.7	26
Pooled	2.4	0.05	1.8	373

*N* refers to the number of data points.

**Figure 5:**
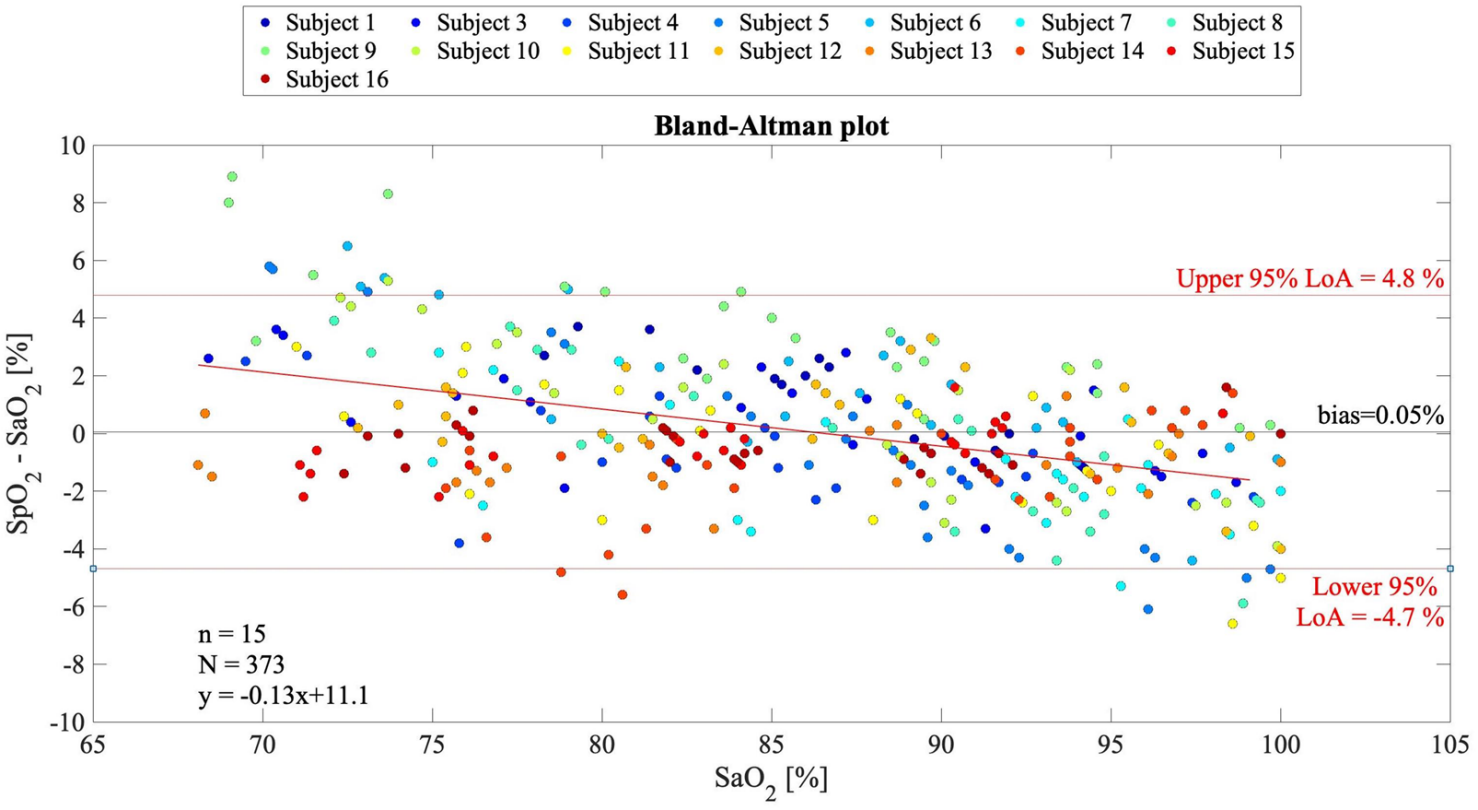
Bland–Altman plot for multiple observations with all subjects pooled (*N* = 373 samples from *n* = 15 subjects) showing the reference SaO_2_ vs. the difference between the EmbracePlus SpO_2_ and the reference SaO_2_. Data from individual subjects are color coded. Bland–Altman linear regression fit (red bold line), upper and lower limits of agreement (thin red lines), and mean bias (black line) are shown. In the bottom right corner, the number of subjects (*n*), data points (*N*), and linear fit equation are reported.

**Figure 6:**
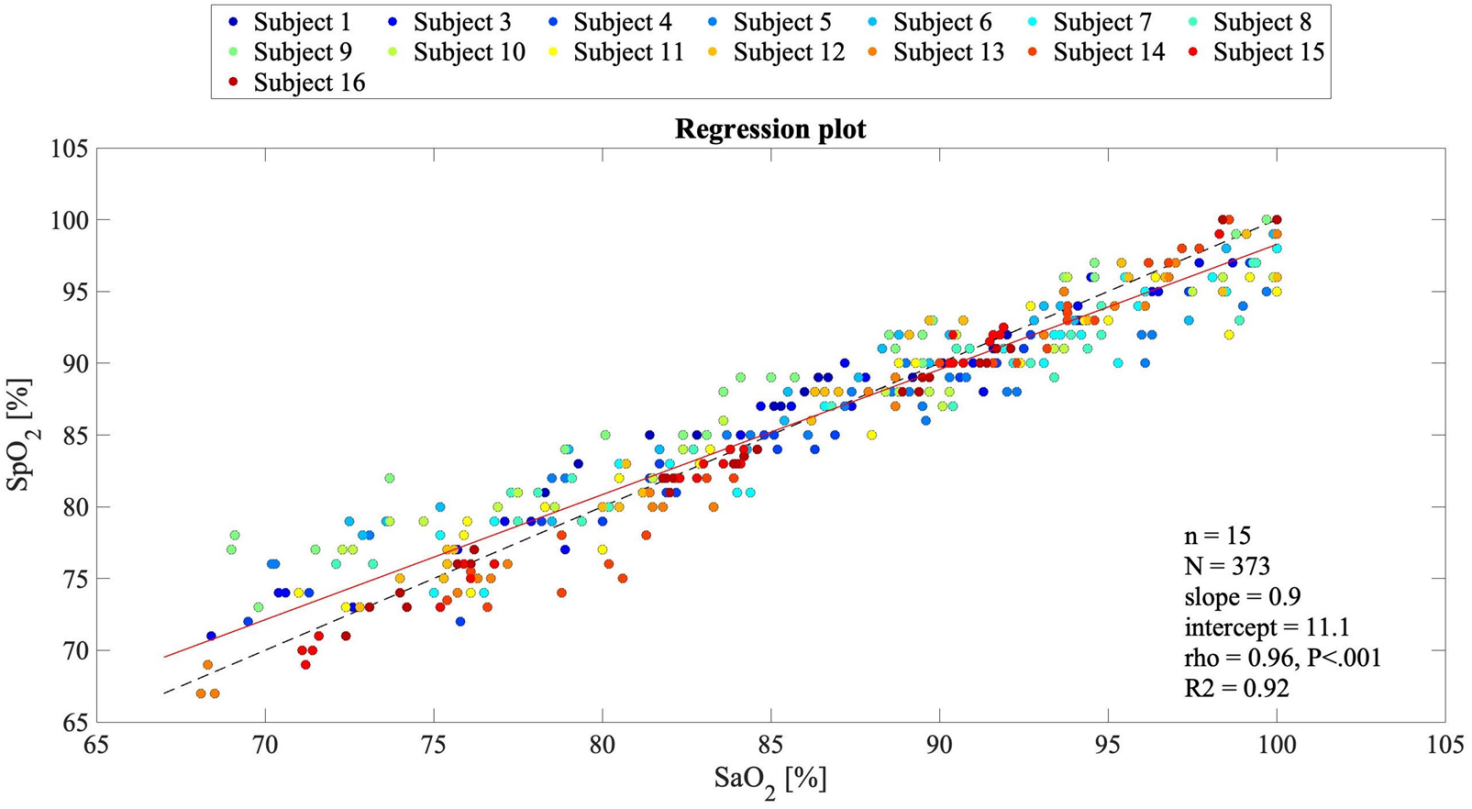
Regression plot for comparing the EmbracePlus SpO_2_ values against the reference SaO_2_ values (*N* = 373 samples from *n* = 15 subjects). Single subject reference SaO_2_ data are plotted against the SpO_2_, with a superimposed bisect line (dashed black line) and a linear regression line (solid red line). In the legend, the slope and the intercept, Pearson's correlation coefficient (rho), its *P* value, and the R2 value of the linear fitting are reported.

**Figure 7:**
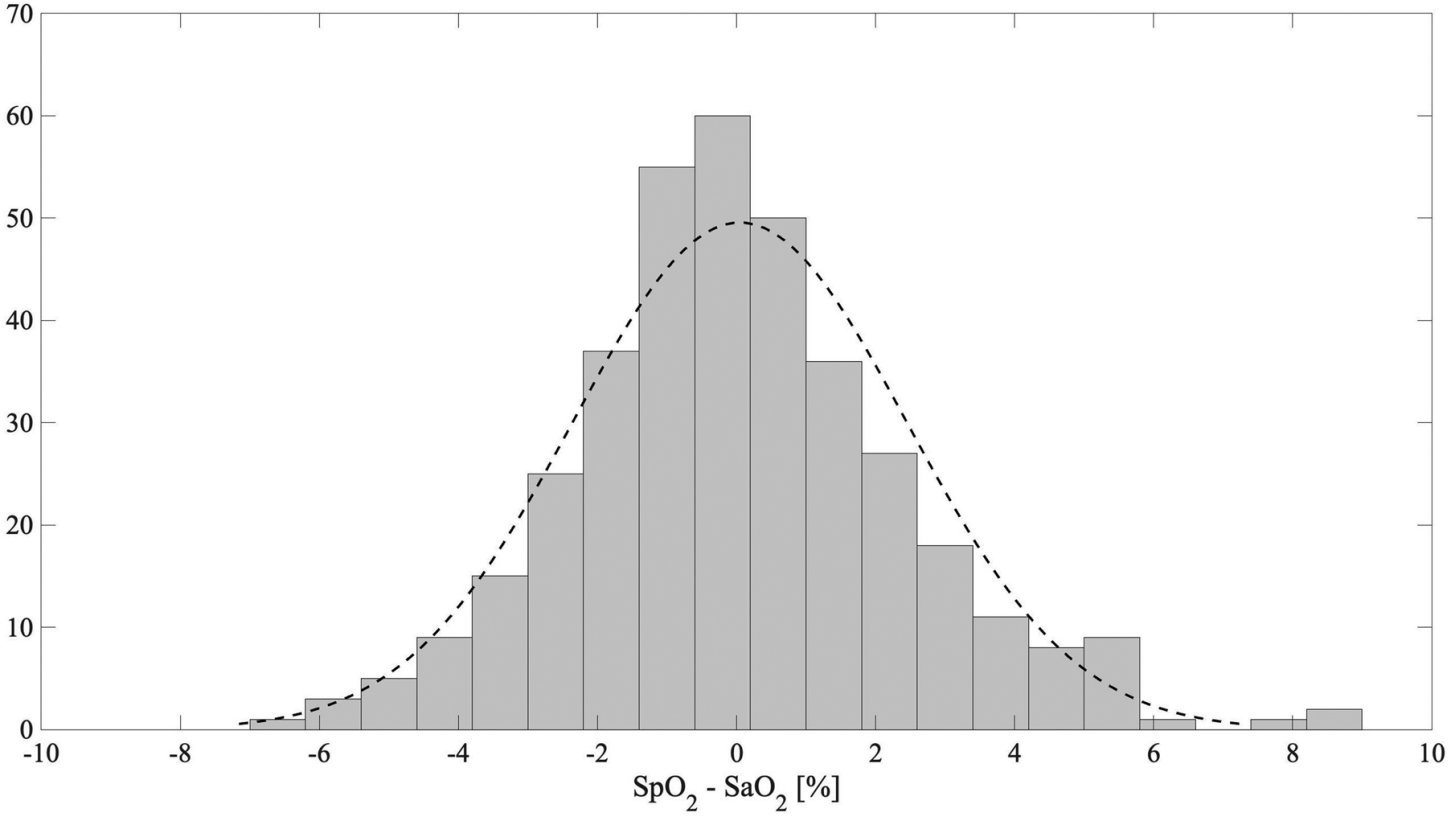
The distribution of the difference between the EmbracePlus SpO_2_ and the reference SaO_2_ (grey) with a normal density function fit (dashed black line).

In Figure 5, the Bland–Altman plot for all the analyzed subjects illustrates the difference between the EmbracePlus SpO_2_ and the reference SaO_2_ with respect to the reference SaO_2_ values. A total of 26 outliers outside the pooled LoAs (i.e., −4.7%; 4.8%) were identified, representing ∼7% of total data points, and are listed in Table 5. Figure 6 reports the regression plot on the pooled data, illustrating a positive, strong correlation (Pearson's correlation coefficient of 0.96) between the SpO_2_ algorithm and the reference SaO_2_. In addition, Figure 7 shows the distribution of the difference between the EmbracePlus SpO_2_ and the reference SaO_2_ measurements with a normal density function fitting.

**Table 5:** List of SpO_2_ samples recognized as outliers.

Subject ID	# blood sample	SaO_2_ [%]	(SpO_2_-SaO_2_) [%]
5	1	99.0	−5
5	3	96.1	−6.1
5	13	73.1	4.9
5	14	99.7	−4.7
5	22	79.0	5.0
5	24	70.3	5.7
5	25	70.2	5.8
6	10	73.6	5.4
6	11	72.5	6.5
6	21	79.0	5.0
6	22	75.2	4.8
6	24	72.9	5.1
7	20	95.3	−5.3
8	1	98.9	−5.9
9	11	80.1	4.9
9	18	84.1	4.9
9	20	78.9	5.1
9	21	73.7	8.3
9	22	69.1	8.9
9	23	71.5	5.5
9	24	69.0	8.0
10	23	73.7	5.3
11	1	98.6	−6.6
11	14	100.1	−5.1
14	13	80.6	−5.6
14	25	78.8	−4.8

For each sample, the subject ID, the corresponding blood sample number, the SaO_2_ value, and the error are reported. Negative error values indicate underestimation by the SpO_2_ algorithm, while positive errors indicate overestimation.

Table 6 reports the SpO_2_ algorithm performance considering skin pigmentation and gender subgroups, as listed in the Statistical Analysis section. Each subgroup analyzed meets or exceeds the FDA-recommended clinical threshold (*A*_rms_ equal or lower than 3.5%). Moreover, comparable LoAs and error metrics were observed between skin and gender subgroups.

**Table 6:** Accuracy metrics of the SpO_2_ algorithm in terms of *A*_rms_, bias, MAE, upper 95% LoA, and lower 95% LoA as described in the statistical analysis section.

Subgroup	Number of subjects	Number of paired samples	Number of paired samples [%]	*A*_rms_ [%]	Bias [%]	MAE [%]	Upper 95% LoA [%]	Lower 95% LoA [%]
Fitzpatrick V–VI	5	129	34.6	2.2	0.1	1.5	4.8	−4.6
Fitzpatrick I–IV	10	244	65.4	2.5	0.04	2.0	4.9	−4.9
Female	8	203	54.4	1.9	−0.5	1.5	3	−4.1
Male	7	170	45.6	2.9	0.7	2.2	6.4	−4.9

Results are derived from the data pooled across dark skin pigmentation (Fitzpatrick V–VI), light skin pigmentation (Fitzpatrick I–IV), female, and male subjects on the [67 100]% reference SaO_2_ range. The percentages of the number of samples are computed with respect to the full dataset, i.e., *N* = 373.

Finally, a non-significant effect of the desaturation ramp on the estimation errors was determined (*p* > 0.05).

## 4. Discussion

### 4.1. Principal results

Wrist-worn wearable devices are increasingly used to monitor SpO_2_ and contribute to promoting home-centered healthcare and decentralized clinical trials (11, 18, 26, 32, 35, 52), with the potential to significantly improve clinical outcomes by supporting prompt diagnosis and early detection of clinical deterioration in patients with respiratory diseases such as COPD, OSA, and COVID-19 (9–12). However, commercially available wearable devices using PPG to measure SpO_2_ are often sold as consumer products rather than medical devices, thereby reducing requirements for evidence of clinical performance and independent review by relevant regulatory authorities (11, 19, 52, 53). Even when cleared as medical devices, concerns have recently emerged about racial bias impacting the performance of pulse oximeters in individuals with darker skin pigmentation (37, 38), highlighting the need for additional data evaluating the reliability of wearable PPG technology.

In this work, we examined the validity of the EmbracePlus wristband reflective pulse oximeter in monitoring SpO_2_ during a controlled hypoxia study. The clinical study was conducted in accordance with the ISO 80601-2-61:2017 standard (15) and FDA guidance (16). Data from sixteen healthy adults were analyzed during a standardized desaturation protocol, and performances of the SpO_2_ EmbracePlus measurements were compared with the gold standard SaO_2_. In this study, we report performance on 373 paired samples from 15 participants, exceeding FDA guidelines on validation of pulse oximeters, which recommend a minimum of 70 data points (i.e., 200/3) in each tested SaO_2_ decile (16). Our dataset included 142, 138, and 93 data points in SaO_2 _≥ 90%, 80% ≤ SaO_2 _< 90%, and SaO_2 _< 80%, respectively. However, given the limited stability of induced desaturations below 80%, the distribution of the collected data is skewed towards values greater than 80%. The *A*_rms_ accuracy between the estimated SpO_2_ and the reference SaO_2_ did meet the passing criterion of *A*_rms_ under 3.5%, reaching a value of 2.4%, indicating that approximately 2/3 of the SpO_2_ values were within ±2.4% of the reference SaO_2_. The effectiveness of the SpO_2_ algorithm was further supported by low bias (<0.5%) and MAE (<2%) and a significantly strong correlation with the reference SaO_2_ (Pearson's correlation coefficient >0.95, *P* < 0.001), suggesting the possibility to track chronic reductions of the average SpO_2_ over time, which could be suggestive of an underlying issue.

Recently, a positive bias and a larger error for SpO_2_ measurements at low blood oxygen saturations in darkly pigmented subjects have been reported by manufacturers of several different pulse oximeters (36, 41), raising concern for potential racial bias (37, 38). Given these concerns, this study enrolled five (i.e., 5/16, ∼31%) subjects with skin pigmentation classified as Fitzpatrick scale V or VI, doubling the FDA requirements of having at least 2 subjects or 15% of the participants (whichever is greater) with dark skin tone (16). The results highlight a global error of 0.1% and 0.04% when considering dark and light skin tones, respectively, with a slightly larger positive bias for darkly pigmented participants but comparable LoAs. Each subgroup analyzed, moreover, was found to have an *A*_rms_ below 3.5% (i.e., Fitzpatrick V–VI, Fitzpatrick I–IV, males, females), indicating acceptable thresholds for accuracy.

An analysis of outliers found a limited number of data points falling outside the pooled Limits of Agreements (i.e., −4.7%; 4.8%), representing <7% of the total. In a risk analysis, the clinical impact of these outliers was determined to be marginal, as no data points fell within a window of occult hypoxemia, defined as a SaO_2_ of <88% with a measured SpO_2_ of 92%–96% (36, 54). For the majority of outliers (17/26, ∼65%), the wearable device produced SpO_2_ overestimates of SaO_2_ for SaO_2_ values ≤84.1%. In each instance, the degree of SpO_2_ overestimation would not change the clinical interpretation of the subject as being critically hypoxic. Nearly all remaining outliers (7, i.e., ∼27%) involved underestimating SaO_2_ values over 95%. These underestimated values remained within the highest SpO_2_ decile, and the bias towards underestimation reduces the risk of occult hypoxemia. Finally, in two cases (i.e., 8%), an outlier was recorded at the upper extreme of the lowest decile (SaO_2_ ≈ 80%) involving underestimation of SaO_2_ within the lowest decile. The degree of SpO_2_ underestimation in these instances would not change the clinical interpretation of critical hypoxia but may bias clinicians toward earlier triage and evaluation. As the device is intended for retrospective data review without alarms or reliance on output for real-time clinical decision-making, the listed outliers do not raise new questions of safety or effectiveness.

The presented results contributed to receiving FDA clearance for the EmbracePlus and its monitoring platform to be used by trained healthcare professionals or researchers to remotely monitor physiological parameters in ambulatory individuals 18 years of age and older in home-healthcare environments (40). The Empatica EmbracePlus wristband is one of the few wearable devices that received 510(k) marketing authorization from the US FDA for SpO_2_ monitoring (21, 22, 27, 28). An analysis of performance data for FDA-cleared wrist-worn SpO_2_ monitoring devices showed comparable levels of overall accuracy, but significant limitations in publicly available data. The Oxitone 1000 M was validated on data recorded during a desaturation protocol similar to that used to test the EmbracePlus algorithm, and the authors reported an *A*_rms_ of 1.9% (27, 35). Given fewer total data points included in the analysis (240 samples from 10 subjects) and a paucity of published data on individuals with darker skin pigmentation (18, 27, 35), the impact of racial bias on performance for this device remains unclear. Similarly, a recent study on 14 subjects wearing the ScanWatch during a similar desaturation protocol of this study showed an accuracy *A*_rms_ of 2.97% (right wrist) and 3% (left wrist) on a comparable population but did not include subgroup analyses evaluating the impact of sex or skin tone on performance (26, 28). Finally, Biobeat Technologies Ltd reports an *A*_rms_ of 2% for their wearable devices; however, to the best of our knowledge, no complete information about the population, number of samples, or performance by subgroups is available (22).

### 4.2. Limitations and future work

The validation presented in this work advances recent efforts from research and public health communities to increase transparency and understanding of the limitations of pulse oximetry. Recommendations that have been put forth to date include publishing subgroup analyses, providing justification for outliers, and increasing the number of data samples analyzed beyond the FDA-suggested sample size (41). Nevertheless, the results of this study must be interpreted in the context of several limitations.

During the hypoxia study, data were collected in a controlled environment with a standardized desaturation protocol to maintain SpO_2_ levels as stable as possible. While the controlled study design fulfills the ISO and FDA guidelines and allows for better assessment of the impact of certain confounders (e.g., demographic variables) on performance through careful experimental control of other covariates, the results do not support analysis of the generalizability of the EmbracePlus wristband to monitor SpO_2_ in real-world conditions, where SpO_2_ exhibits dynamic changes over time.

Additionally, the study pool was limited to healthy subjects aged 18–43 years, due to higher risks induced by hypoxia in older and/or unhealthy patients. There are an increasing number of investigations evaluating the performance of wrist-worn devices in uncontrolled studies and/or on patients with cardiovascular and lung diseases, yet these are mostly performed with consumer smartwatches thanks to their wider availability and lower costs (11, 19, 32, 52). As there are not rigorous evaluation requirements for SpO_2_ computation in these consumer products, and they do not go through independent regulatory review, however, it is unclear to what degree clinical study results using these products can be relied upon to understand the impact of race, comorbidities, or real-world conditions on medical device performance (25).

**Figure 8:**
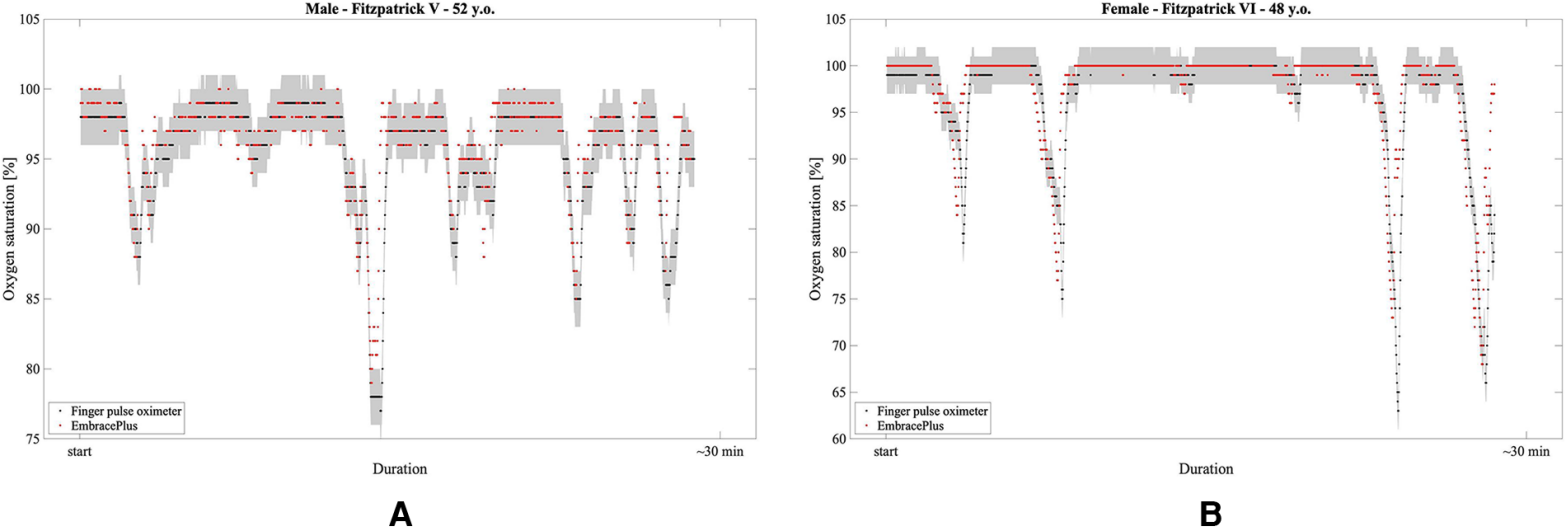
Examples of SpO_2_ measurements during hyperventilation-induced desaturations: in red, the 1 s EmbracePlus SpO_2_ outputs; in black, the finger pulse oximeters samples; in grey, the clinical acceptance boundaries for finger pulse oximeter of ±2%. (**A**) 52 years male subject with Fitzpatrick V skin pigmentation; (**B**) 48 years female subject with Fitzpatrick VI skin pigmentation.

Following promising preliminary data collected during a separate, ambulatory, uncontrolled desaturations in darkly pigmented adults (Figure 8), future work will focus on extensively testing the EmbracePlus wristband in real-life conditions (e.g., sleep), in diverse age ranges, and on specific pathological conditions (e.g., lung diseases like COPD and obstructive sleep apnea). Future investigations may assess the feasibility of using the SpO_2_ data not only to detect hypoxemia but also to assess cardiopulmonary function [e.g., track SpO_2_ recovery after exercise (55)] or for stress monitoring (56).

## 5. Conclusions

To conclude, the study results demonstrate that the SpO_2_ measurements performed by the non-invasive EmbracePlus wristband show high clinical accuracy between 70%–100% SpO_2_ in the intended use conditions of no-motion and high-perfusion, across individuals with a range of skin pigmentation. This study contributes to the current state of scientific knowledge on the impact of racial bias on SpO_2_ measurement and paves the way for further validation during prolonged use in uncontrolled settings and on patients at risk of hypoxemia.

## Data Availability

The datasets presented in this article are not readily available because the sensor data recorded on the patients are proprietary of Empatica Inc. The authors can share the data on arterial oxygen saturation and the output of the SpO_2_ algorithm for each patient upon request to the corresponding author. Requests to access the datasets should be directed to gg@empatica.com.
